# The first dog-origin porcine circovirus type 4 complete genomic sequence have high homology with that of pig-derived strains

**DOI:** 10.3389/fmicb.2023.1121177

**Published:** 2023-02-24

**Authors:** Tong Xu, Lan Chen, Bing-Zhou Huang, Ling Zhu, Xian-Gang Sun, Si-Yuan Lai, Yan-Ru Ai, Yuan-Cheng Zhou, Zhi-Wen Xu

**Affiliations:** ^1^College of Veterinary Medicine, Sichuan Agricultural University, Chengdu, China; ^2^Sichuan Key Laboratory of Animal Epidemic Disease and Human Health, College of Veterinary Medicine, Sichuan Agricultural University, Chengdu, China; ^3^Key Laboratory of Animal Breeding and Genetics Key Laboratory of Sichuan Province, Sichuan Animal Science Academy, Chengdu, China; ^4^Livestock and Poultry Biological Products Key Laboratory of Sichuan Province, Sichuan Animal Science Academy, Chengdu, China

**Keywords:** porcine circovirus 4, dog, molecular detection, genetic analysis, cross-species transmission

## Abstract

**Introduction:** Porcine circovirus 4 (PCV4) was discovered in 2019 and then proved to be pathogenic to piglets. Nevertheless, few studies were currently available about PCV4 infection in species other than pigs and there is no information about the prevalence of PCV4 in dogs.

**Methods:** To fill this gap, 264 dog samples were collected from animal hospitals in the Southwest of China from 2021 to 2022 and screened for PCV4. Moreover, the complete genome of one PCV4 strain (SCABTC-Dog2022) were obtained successfully and shared a high identity (97.9-99.0%) with other PCV4 strains derived from pigs, dairy cows, raccoon dogs and foxes. The SCABTC-Dog2022 were analyzed together with 51 reference sequences.

**Results and Discussion:** The detected results showed a low percentage of PCV-4 DNA (1.14%, 3/264), indicating that PCV4 could be identified in dogs in southwest China. Phylogenetic tree showed that SCABTC-Dog2022 strain derived from dog were clustered in a closed relative and geographically coherent branch with other PCV4 strains collected from four provinces (Sichuan, Fujian, Hunan and Inner Mongolia) of China. To our knowledge, it is the first detection of PCV4 in dogs globally. The association between PCV4 status and clinical syndromes in dogs deserves additional investigations.

## 1. Introduction

Porcine circoviruses (PCVs) are members of family *Circoviridae*, genus *Circovirus*, and also the smallest envelope-free animal DNA viruses (Opriessnig et al., 2020). Up to date, at least four PCVs have been recognized with similar structure, including porcine circovirus 1 (PCV1), porcine circovirus 2 (PCV2), porcine circovirus 3 (PCV3), and porcine circovirus 4 (PCV4). The genome of PCVs is circular single-stranded DNA and ranging from about 1.7 to 2.1 kb in size (Lekcharoensuk et al., 2004; Cheung, 2012; Opriessnig et al., 2020).

PCV1 was deemed to be non-pathogenic to pigs and was first reported in 1982 (Tischer et al., 1974, 1986; Allan et al., 1995). In the 1990s, PCV-2 was identified experimentally as the cause of porcine circovirus-associated disease (PCVAD) in Canada, followed by severe outbreaks worldwide, which resulted in important losses to the pig production (Nayar et al., 1997; Allan et al., 1998; Ellis et al., 1998; Kiupel et al., 1998; Morozov et al., 1998). Porcine circovirus disease (PCVAD) was a collective term for systemic and reproductive diseases, including post-weaning multisystemic wasting syndrome (PMWS), porcine dermatitis and nephrotic syndrome (PDNS), reproductive disorders, and respiratory diseases (Nayar et al., 1997; Allan et al., 1998; Ellis et al., 1998; Kiupel et al., 1998; Morozov et al., 1998). Porcine circovirus 3 (PCV-3) was discovered in the United States in 2015. Then, it has been detected globally from pigs with several clinical signs including respiratory disease, enteritis and PDNS (Phan et al., 2016; Palinski et al., 2017). The emerging porcine circovirus type 4 (PCV4) was discovered in pigs displaying severe clinical pathological outcomes as well as in apparently healthy pigs in 2019 in Hunan, China (Zhang D. et al., 2020), and then reported in several provinces in China as well as in South Korea (Chen et al., 2021; Sun et al., 2021; Tian et al., 2021; Kim et al., 2022; Xu et al., 2022a). Recently, Niu et al. successfully rescued PCV4 from an infectious clone and demonstrated its pathogenicity to piglets (Niu et al., 2022).

Besides the natural reservoir of pigs, PCVs has also been identified in non-porcine animals. In 1995, the presence of PCV1 antibodies in cattle was first reported in Germany (Tischer et al., 1995). Subsequently, PCV2 DNA was found in cattle with respiratory disease and from aborted bovine fetuses. In addition, the PCV2 genome has been reported to be present in a variety of non-pig animals, such as foxes, rats, dogs etc (Kiupel et al., 2001; Herbst and Willems, 2017; Song et al., 2019a). PCV3 DNA were also detected in non-porcine animals, such as ruminants, rodents and canines, etc (Zhang et al., 2018; Jiang et al., 2019; Wang et al., 2019). Available data indicate that PCVs (PCV1–PCV3) have a broad host spectrum. Moreover, PCVs cross-species transmission is likely to be a serious threat to the global pig industry and other animal industries (Turlewicz-Podbielska et al., 2022). However, there were few studies on PCV4 infection in species other than pigs. The cross-species transmission of PCV4 should be paid more attention.

Whether dog was one of the hosts of PCV4 remained unknown. Based on this premise, the present study aimed to investigate the presence and circulation of PCV4 in dogs. Then, 264 dog samples were collected and detected for the presence of PCV4, and the complete genome was sequenced.

## 2. Materials and methods

### 2.1. Clinical samples collection and viral DNA extraction

A total of 264 clinical samples (fecal samples, rectal swabs, and nasal swabs) from dogs with several clinical manifestations (respiratory disease and diarrhea) were collected from 15 animal hospitals in 9 cities (Mianyang, Suining, Chengdu, Deyang, Luzhou, Dazhou, Guangan, Guangyuan, and Yibin) in the southwest of China during 2021–2022. The samples were dissolved in an Eppendorf tube containing phosphate-buffered saline (pH 7.2). The homogeneity was either used immediately for DNA extraction or stored at −80°C until use.

DNA was extracted using the Universal Genomic DNA Kit (CoWin Biotech Co, Ltd., Taizhou, China) in accordance with the manufacturer’s instructions. To detected PCV4, a SYBR Green І-based qPCR assay was performed as described previously (Xu et al., 2022a).

### 2.2. Complete genome sequencing

The positive samples were selected for the complete genome amplification of PCV4 as described previously (Xu et al., 2022b). Three overlap primer pairs for amplifying the whole genome are described in Supplementary Table S1. In brief, the PCR reaction mixture was performed in a 50 μL total reaction containing 25 μL of 2 × Phanta Flash Master Mix (Nanjing Vazyme Biotech Co., Ltd. Nanjing, China), 1 μL (25 μM) of each pair of primers, 4 μL of viral DNA, and 19 μL of ddH_2_O. The amplification was performed with initial incubation at 98°C for 30 s; 35 cycles of 10 s at 98°C, 60°C for 10 s, and 72°C for 5 s, and a final extension for 10 min at 72°C. The overlapping DNA fragments were sent to Tsingke Biotechnology Co., Ltd., Beijing, China for sequencing.

### 2.3. Sequence alignment and phylogenetic analysis

The dog-origin PCV4 strain in this study was further analyzed with 51 PCV4 strains all available in GenBank database (accessed 22 October 2022). All available information regarding reference strains is provided in Supplementary Table S2. The DNAstar software (DNAStar V7.1, Madison, WI, United States) was used for assembly and alignment. A neighbor-joining (NJ) phylogenetic tree was constructed with a p-distance model, and a bootstrap of 1,000 replicates using Molecular Evolutionary Genetics Analysis (MEGA) software (version 7.0).

## 3. Results and discussion

PCVs (PCV1-3) could propagated in hosts other than pigs and were associated with several clinical signs under field conditions (Song et al., 2019a,b). Herbst and Willems (2017) detected PCV2 genome in feces of dogs in Germany (Herbst and Willems, 2017). Likewise, Sun et al. (2021) reported PCV3 DNA in serum samples of dogs and PCV3-positive rate was 23.6% (96/406) (Sun et al., 2019). Nevertheless, it is still unknown whether dog is one of the reservoirs of PCV4. The objectives of this study were to investigate the presence of PCV4 in dog and to perform further DNA sequencing analysis.

**Figure 1:**
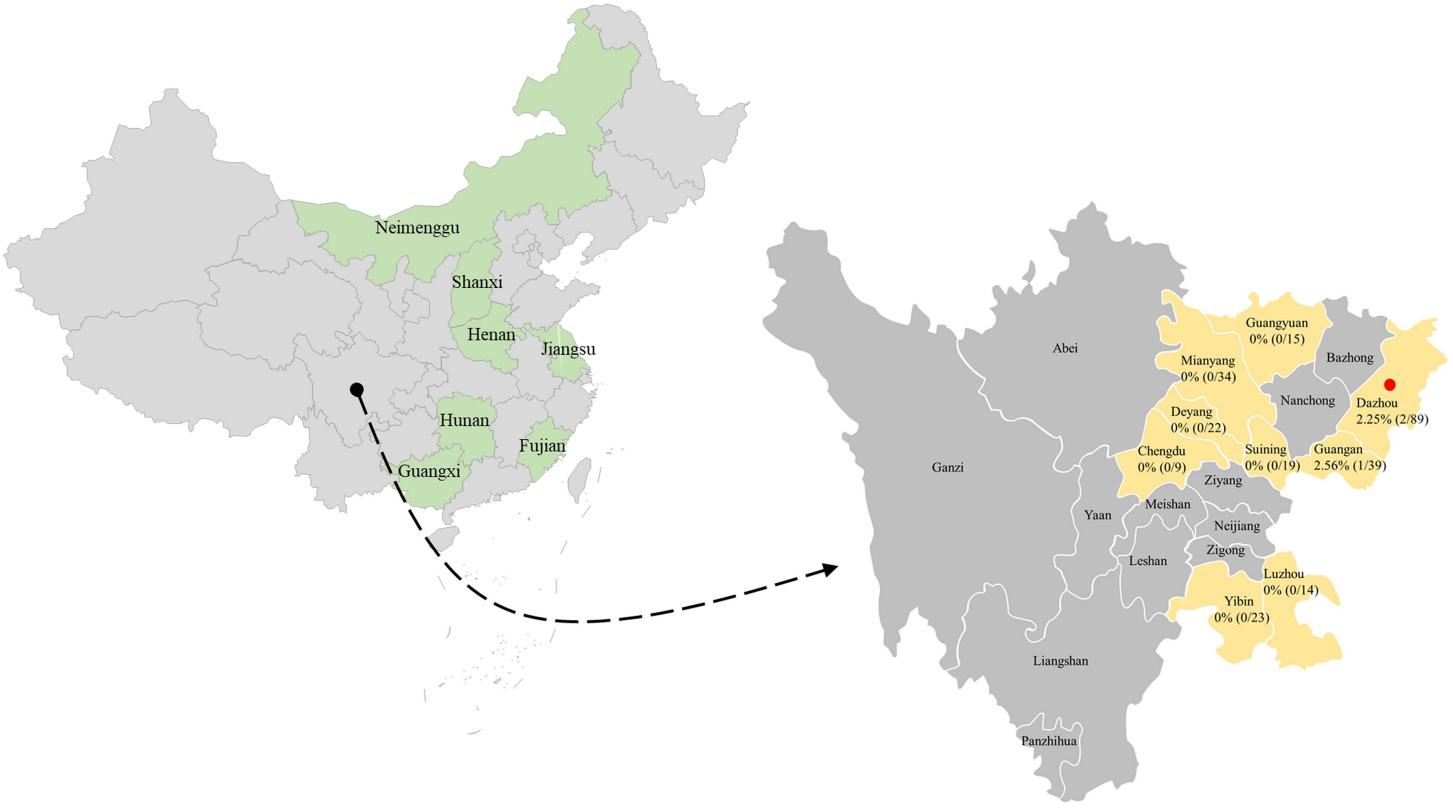
The geographical distribution of the 264 samples. In China, the provinces with PCV4 identification were filled with light green. The clinical samples in this study were collected from light gold-filled cities in southwestern China, and cities with positive samples were marked by red solid circles. The numbers indicate the positivity rate of PCV4 in different cities.

In this epidemiological work, a total of 264 clinical samples from dogs at various growth stages were collected from selected clinically unhealthy animals with clinical disease including respiratory signs and enteric signs. PCV4 DNA was detected in 3 out of 264 tested clinical samples (1.14%) in the present study (Figure 1), which was close to the prevalence found in clinical samples derived from pigs in the same geographical location (Southwest China) in a previous study (Xu et al., 2022c). PCV4 was first reported in pig farms in Hunan province, China in 2019 with a high prevalence of 12.8%, and in another study in Henan and Shanxi Provinces even higher, showing a frequency of 25.40% (16/63) in pig farms (Zhang H. H. et al., 2020; Tian et al., 2021). Interestingly, 3 positive samples were collected from two animal hospitals in Dazhou, Southwest China in 2022, and in our previous study in the same city positive samples from pig farms was also detected, showing diarrheal symptoms co-infected with PEDV. Besides diseased pigs with clinical signs, the PCV4 genome was also detected in healthy pigs (Zhang H. H. et al., 2020). Subsequently, PCV4 was rescued from an infectious clone and pigs inoculated with rescued PCV4 showed no obvious clinical symptoms under experimental infections, while obvious pathological changes in several organs of piglets inoculated with PCV4 suggested that it was pathogenic to piglets (Zhang H. H. et al., 2020). The clinical samples in this study were all from diseased dogs in animal hospitals, and the prevalence of PCV4 in healthy dogs and its pathogenicity in dogs need further study. To date, PCV4 has been identified in several provinces in China and Korea, with positivity rates ranging from 1.34% to 45.39% (Zhang H. H. et al., 2020; Sun et al., 2021; Hou et al., 2022; Nguyen et al., 2022; Xu et al., 2022a,c), but no evidence of PCV-4 presence was found in Italy, Spain, and Colombia through two exploratory studies (Franzo et al., 2020a; Vargas-Bermudez et al., 2022). Anyway, the most likely reason for the different positive rates among published studies could be the species of animals, health status, different geographical locations, epidemiology of each period, and detection methods used. Two different studies recently identified PCV4 DNA in wild boar and dairy cattle in Jiangsu and Henan, respectively (Wu et al., 2022; Xu et al., 2022b), and the whole genome of PCV4 from fur animals (fox and raccoon dogs) in Hebei Province could also be found in GenBank database without available corresponding published studies in the PubMed database. In addition, 2 out of 3 positive samples were nasal swabs and 1 was fecal sample. Notably, one serum was collected from one of three PCV4-positive dogs, and PCV4 DNA was also detected in the serum. The serum was not described in detail in the “Materials and methods” section because it was collected from the same dog along with a fecal sample. This is the first time to provided supported evidence of PCV4 prevalence in dogs in the southwest of China.

Host jumping of circovirus could be a potential threat to public health (Turlewicz-Podbielska et al., 2022). Regarding PCV1 and PCV2, replication was observed in human 293 and Hep2 cells (Hattermann et al., 2004), and antibodies against PCV1 or PCV2 could be detected in human serum, GI samples and respiratory samples (Bernstein et al., 2003; Li et al., 2010; Borkenhagen et al., 2018). It is unclear whether PCV4 has zoonotic potential, if so, whether dogs, as human companion animals, play an important role in the transmission of PCV4 between humans and animals. Much attention should be paid to the abovementioned premises.

In this study, there were three PCV4 positive samples, of which two PCV4 whole genomes obtained from two dog samples collected from animal hospital in Dazhou in 2022, including one nasal swab and one fecal sample. However, another positive sample failed to be amplified. It is very likely that the low viral load of another positive sample may account for failure of full-genome amplification. Among which one unique sequence (SCABTC-Dog2022) were generated and deposited in the GenBank database under the following accession number (OP948894). The full-genome of SCABTC-Dog2022 strain was 1,770 nt in length without deletions and insertions of nucleotides and further analyzed together with 51 PCV4 strains all available in GenBank database (accessed 22 October 2022).

Pairwise-sequence comparisons based on nucleotide sequences indicated the SCABTC-Dog2022 shared 97.9%–99.0% identity of complete genome with 51 PCV4 reference strains. Among which 5 reference strains were sequenced from raccoon dogs, 1 was sequenced from fox, 2 were sequenced from wild boars, and others were sequenced from domestic pigs. In addition, the 51 PCV4 reference strains were from different provinces in China and South Korea, and when the data was analyzed by the year, some strains came from the clinical samples that have been preserved for 10 years. These results suggested that PCV4 can be transmitted across species and regions and dates back at least a decade, and available reference strains from different geographical locations and species at different times shared high similarity of complete genome. Compared to PCV2 with high genomic variation, PCV4 shared higher similarity among available sequences, indicated a slower evolutionary rate of PCV4.

**Figure 2:**
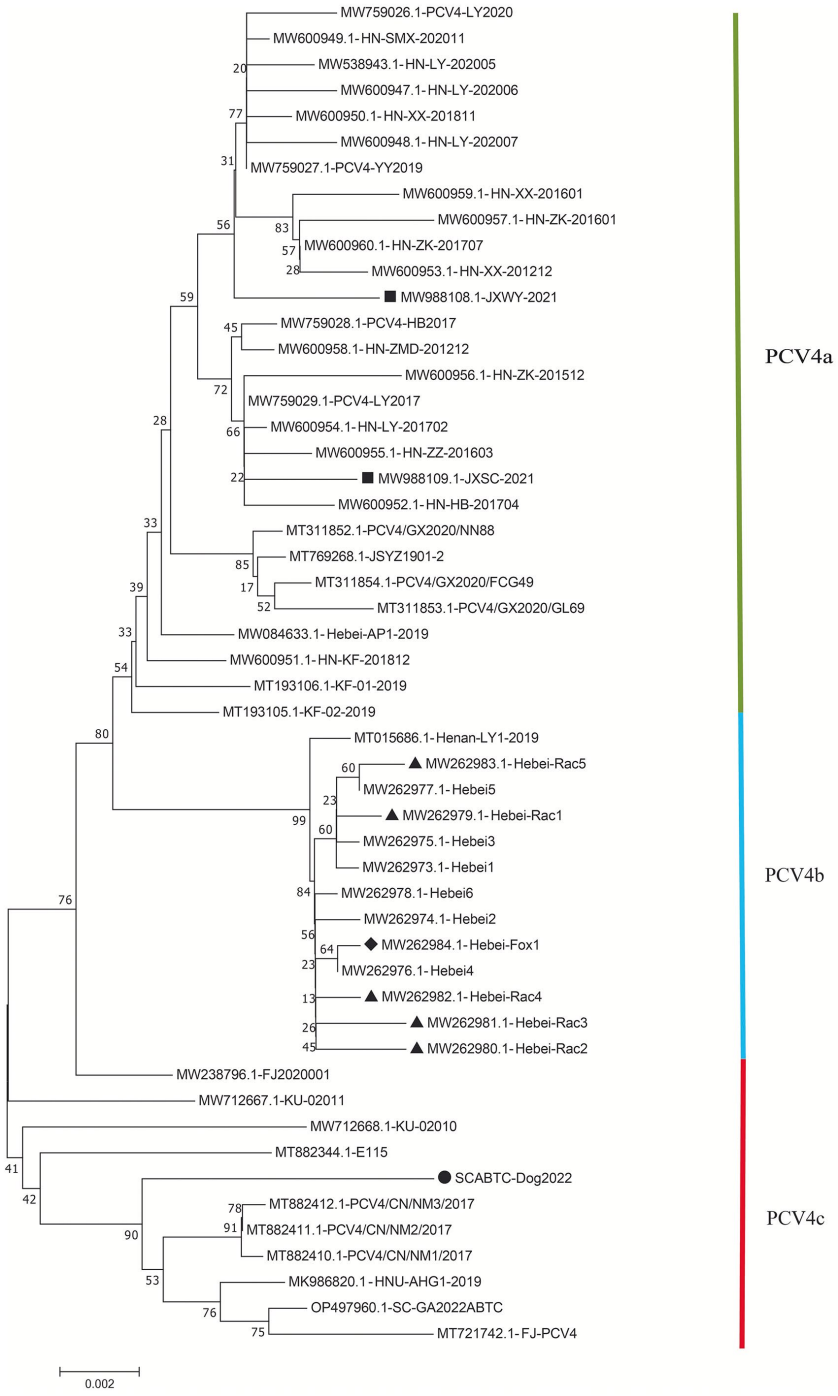
Neighbor-joining trees were constructed with a p-distance model and bootstrapping at 1,000 replicates. Phylogenetic tree was constructed based on the complete genome of 52 PCV4 strains. The black solid triangle (▲) represented the PCV4 strains derived from raccoon dogs. The solid black diamond (◆) represented the fox-derived PCV4 strains. The solid black square (■) represents the PCV4 strains originating from wild boars. The black solid circle (●) represented the dog-derived PCV4 strain (SCABTC-Dog2022) in this study. All unlabeled strains were from domestic pigs. The subtypes were proposed by Xu et al. (2022a).

The high similarity between the available sequences makes it difficult to determine further genotypic classification of PCV4, similar to that of PCV3. Although it is still too early for a definitive classification, two (PCV-4a and PCV-4b) or three major clusters (PCV4a, PCV4b and PCV4c) have been temporarily proposed for this virus (Xu et al., 2022a,b). The NJ phylogenetic tree was established based on the clade classification and amino acid marker positions proposed by Xu et al. (2022b). Phylogenetic analysis showed that SCABTC-Dog2022 clustered with nine pig-origin reference strains in the proposed PCV4c, with three reference strains from Korea and six from three provinces (Fujian, Hunan, and Inner Mongolia) in China. PCV4a was comprised of 28 strains, two of which were from wild boars in Jiangsu Province, China, and others were from domestic pigs in four Chinese provinces (Jiangsu, Guangxi, Henan, and Hebei). In addition, 13 strains belonged to PCV4b, one of which was from domestic pig in Henan province and others were from fur animals (fox and raccoon dog) and domestic pig in Hebei. Geographically, Henan Province and Hebei Province are adjacent to each other. However, when a larger number of sequences were used, the current classification in Figure 2 was inconsistent with previous studies, indicating the necessity of a more reasonable classification scheme. The specific amino acid patterns for PCV4a (239 V in Rep, 27S, 28R, and 212 L in Cap) and PCV4b (239 L in Rep, 27S, 28G and 212 L in Cap) in this study were consistent with those described by Xu et al. (2022b). Compared to marker positions for PCV4a (239 V for Rep protein, 27 N, 28R, and 212 M for Cap protein), two strains (KU-02010 and KU-02011) in PCV4a contained an amino acid mutation (N27S) in Cap and one strains (FJ2020001) had an amino acid mutation (V239L; Xu et al., 2022b), suggesting that the proposed amino acid marker positions were not suitable for determining clade divisions. With the increase of sequences used, the classification of some strains in different studies was not consistent. The high homology of all available PCV4 sequences at the whole genome level does not support further classification of PCV4. Genetic distance and phylogenetic clustering should be the primary objective criteria, and other factors, including the number of sequences within clusters, host and geographic clustering, concordance between different genomic regions, and analytical methods are also taken into account to generate a classification that can be used effectively for research and diagnosis (Franzo et al., 2020b). Therefore, it is too early to classify PCV4 due to the limited sequence information, and because of this more, we encourage more research teams to upload annotated sequences from different geographical locations and different species in a free database.

**Figure 3:**
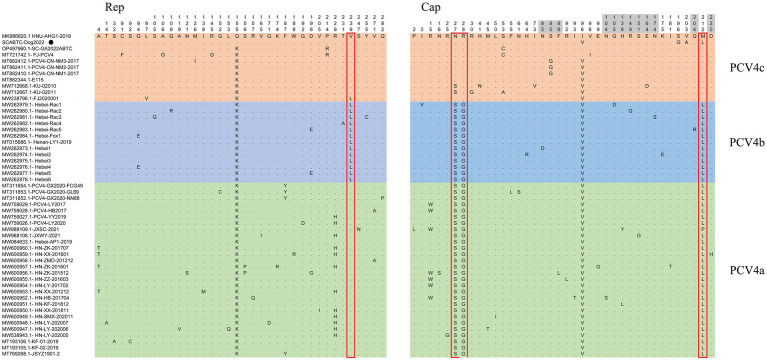
Sequence alignment analysis indicated that there were 36 and 38 amino acid mutations in the Rep and Cap of 52 PCV4 strains. The light green, light blue and light orange colors indicated PCV4a, PCV4b, and PCV4c proposed by Xu et al. (2022b). The red open box showed the specific amino acid patterns of genotypes. Amino acid residues in gray areas contained represented in potential linear B-cell epitopes.

**Figure 4:**
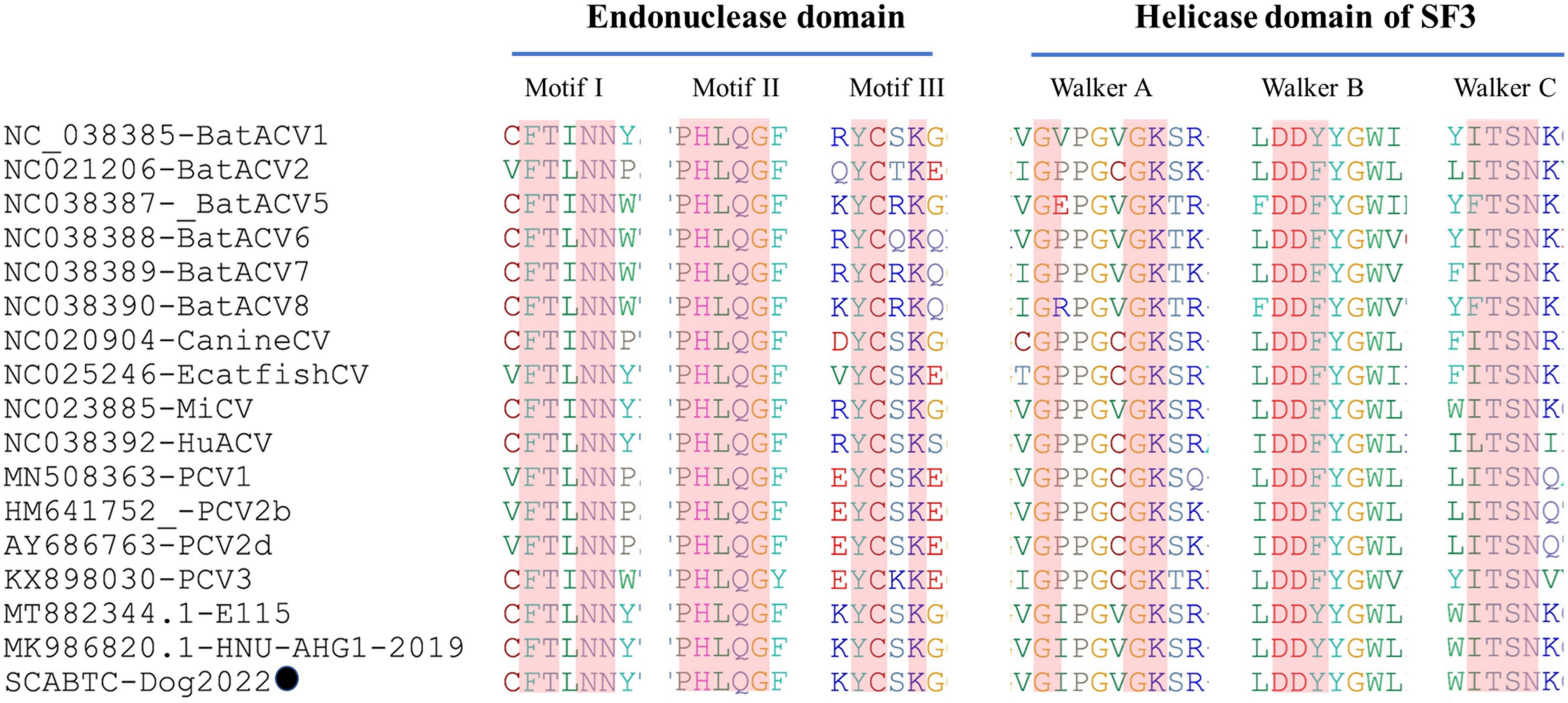
The N-terminal endonuclease domain and the helicase domain of superfamily 3 (SF3) described by Nguyen et al. (2022), were also observed in SCABTC-Dog2022. proposed by Fux et al. The black solid circle (●) represented the dog-derived PCV4 strain (SCABTC-Dog2022) in this study.

Sequence analysis based on 52 PCV4 strains indicated that 36 and 38 amino acid mutations were observed in the Rep and Cap, respectively (Figure. 3). All mutation sites were non-synonymous mutations. The resulting effects warranted further study. Regarding Rep, the N-terminal endonuclease domain including three conserved motifs (motifI−^13^FTLNN^17^, motifII−^50^PHLQG^54^ and motifIII−^90^YCSK^93^) and the helicase domain of superfamily 3 (SF3) containing three Walker motifs (Walker A-^168^GxxxxGKS^175^, Walker B-^207^DDY^209^, and Walker C-^245^ITSN^248^), describing in pig-origin strains (Nguyen et al., 2022), were also observed in SCABTC-Dog2022 (Figure 4). In this study, these functional regions were highly conserved among different PCV4 strains.

**Figure 5:**
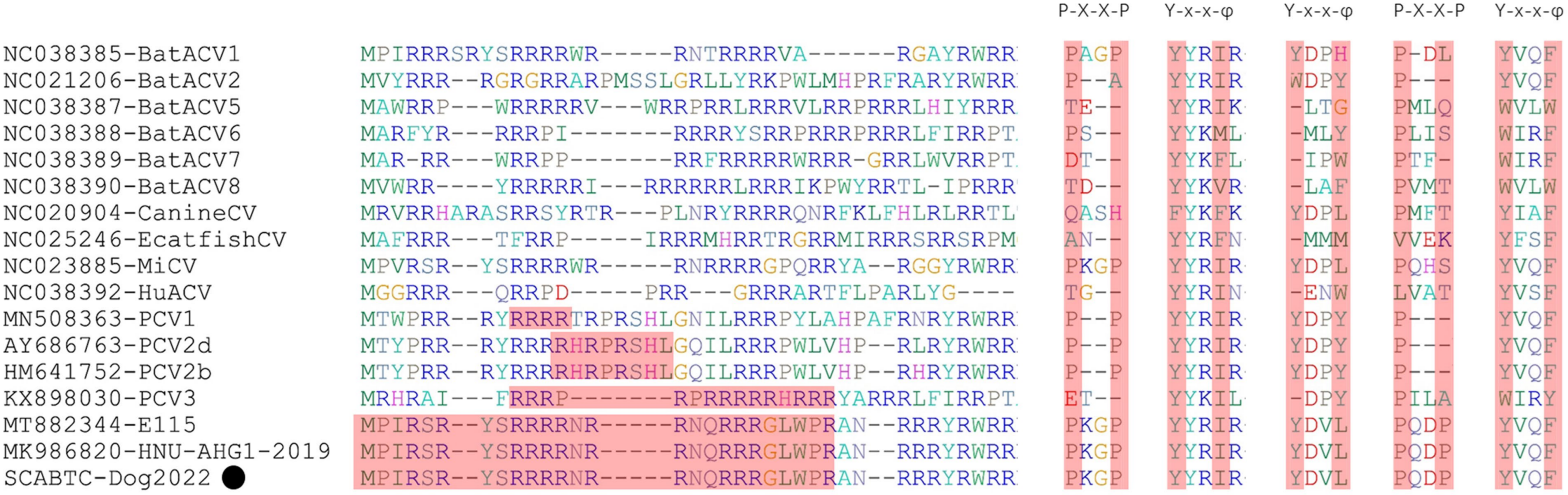
The NLS regions of PCVs (PCV1-4) were highlights in red and reported in several studies (Liu et al., 2001; Shuai et al., 2008; Mou et al., 2019; Wang et al., 2021). The P-x-x-P and Y-x-x-φ patterns were first identified in other viruses belonging to the genus Circovirus. However, the second P-x-x-P was only detected in PCV4 strains (Nguyen et al., 2022). The “x” indicates any amino acid, and “φ” represents any of F (phenylalanine), I (isoleucine), L (leucine), or V (valine). The black solid circle (●) represented the dog-derived PCV4 strain (SCABTC-Dog2022) in this study.

For the Caps of the other three PCVs (including PCV1, PCV2, and PCV3), the nuclear localization signal (NLS) region is an arginine-rich region within the genus circovirus that mediates nuclear targeting of the viral genome and has been experimentally confirmed (Liu et al., 2001; Shuai et al., 2008; Mou et al., 2019). Moreover, a recent study confirmed the NLS region in the N-terminus of PCV4 Cap, ranging from 1 to 20 amino acid (Zhou et al., 2021), which was also observed in Cap of SCABTC-Dog2022 without amino acid mutations. Among PCV4a strains, seven Chinese strains, including one strain (JXSC-2021) derived from Jiangxi Province and six strains (PCV4-LY2017, PCV4-HB2017, HN-ZK-201512, HN-ZZ-201603, HN-LY-201702, and HN-HB-201704) derived from Henan Province, had a characterized amino acid mutation (R15W) in the Cap protein (Figure 3). Besides, two amino acid mutations (P2L and I3V) were found in Hebei-Rac1 strain and JXSC-2021 strain, respectively. The amino acid mutation sites R15W, P2L, and I3V were found to occur in the NLS region of the Cap protein. Whether these amino acid mutations have any effect on the function of NLS deserves further study. Two patterns (P-x-x-P and Y-x-x-φ) are associated with lectin-mediated endocytosis and have been proven to play an essential role in the host entry mechanism of *Circoviridae* (Wei et al., 2018), which was recently described in PCV4 strains from pigs (Nguyen et al., 2022) as well as in SCABTC-Dog2022 sequenced in this study (Figure 5). Five potentially linear B-cell epitope regions with high antigenicity were predicted in a previous study, including amino acid positions 72–88, 104–112, 122–177, 199–205, and 219–225 (Wang et al., 2021). Out of 38 amino acid mutations in of Cap of 52 PCV4 strains, 12 were located in the predicted epitope region, 12 were located in the predicted epitope region, which may have altered the antigenicity of PCV4 Cap, but the potential immunogenicity changes due to amino acid mutations in the Cap epitope region need to be demonstrated by further studies.

## 4. Conclusion

To our knowledge, this is the first time that the genome of PCV4 has been identified in dogs. The first dog-origin complete genome sequence (SCABTC-Dog2022) shared a high homology (>97.9%) with 51 PCV4 reference strains derived from different species in different geographical locations, indicating a slow evolutionary rate of PCV4. The infectious mechanism and pathogenicity of PCV4 in dog merits further study.

## Data availability statement

The datasets presented in this study can be found in online repositories. The names of the repository/repositories and accession number(s) can be found in the article/Supplementary material.

## Ethics statement

The types of field samples used in this study included fecal samples, rectal swabs, and nasal swab. This article does not contain any studies with human participants or animals performed by any of the authors.

## Author contributions

X-GS, S-YL, and LZ contributed to conceptualization. Y-CZ and Y-RA contributed to the methodology. TX contributed to software and writing—original draft preparation. LC and B-ZH contributed to validation and supervision. Z-WX contributed to project administration and funding acquisition. All authors contributed to the article and approved the submitted version.

## Funding

This work was supported by the Chongqing Municipal Technology Innovation and Application Development Project (no. cstc2021jscx-dxwt BX0007), the Key K&D Program of Sichuan Science and Technology Plan (no. 2022YFN0007), the Porcine Major Science and Technology Project of Sichuan Science and Technology Plan (no. 2021ZDZX0010-3), the Sichuan Provincial Department of Science and Technology Rural Area Key R&D Program (no. 2020YFN0147), the Agricultural Industry Technology System of Sichuan Provincial Department of Agriculture (No. CARSSVDIP), Science and Technology Program of Sichuan Province (No. 2022YFQ0023), and Science and Technology Program of Sichuan Province (No. 2022YFN0069).

## Conflict of interest

The authors declare that the research was conducted in the absence of any commercial or financial relationships that could be construed as a potential conflict of interest.

## Publisher’s note

All claims expressed in this article are solely those of the authors and do not necessarily represent those of their affiliated organizations, or those of the publisher, the editors and the reviewers. Any product that may be evaluated in this article, or claim that may be made by its manufacturer, is not guaranteed or endorsed by the publisher.

## Supplementary material

The Supplementary material for this article can be found online at: https://www.frontiersin.org/articles/10.3389/fmicb.2023.1121177/abstract#supplementary-material

## References

[ref1] Allan G. McNeilly F. Cassidy J. Reilly G. Adair B. Ellis W.. (1995). Pathogenesis of porcine circovirus; experimental infections of colostrum deprived piglets and examination of pig foetal material. Vet. Microbiol. 44, 49–64. doi: 10.1016/0378-1135(94)00136-K 7667906

[ref2] Allan G. McNeilly F. Kennedy S. Daft B. Clarke E. Ellis J.. (1998). Isolation of porcine circovirus-like viruses from pigs with a wasting disease in the USA and Europe. J. Vet. Diagn. Invest. 10, 3–10. doi: 10.1177/104063879801000102, PMID: 9526853

[ref3] Bernstein C. N. Nayar G. Hamel A. Blanchard J. F. (2003). Study of animal-borne infections in the mucosas of patients with inflammatory bowel disease and population-based controls. J. Clin. Microbiol. 41, 4986–4990. doi: 10.1128/JCM.41.11.4986-4990.2003, PMID: 14605128 PMC262476

[ref4] Borkenhagen L. K. Mallinson K. A. Tsao R. W. Ha S. J. Lim W. H. Toh T. H.. (2018). Surveillance for respiratory and diarrheal pathogens at the human-pig interface in Sarawak, Malaysia. PLoS One 13:e0201295. doi: 10.1371/journal.pone.0201295 30052648 PMC6063427

[ref5] Chen N. Xiao Y. Li X. Li S. Xie N. Yan X.. (2021). Development and application of a quadruplex real-time PCR assay for differential detection of porcine circoviruses (PCV1 to PCV4) in Jiangsu province of China from 2016 to 2020. Transbound. Emerg. Dis. 68, 1615–1624. doi: 10.1111/tbed.13833, PMID: 32931644

[ref6] Cheung A. K. (2012). Porcine circovirus: transcription and DNA replication. Virus Res. 164, 46–53. doi: 10.1016/j.virusres.2011.10.012, PMID: 22036834

[ref7] Ellis J. Hassard L. Clark E. Harding J. Allan G. Willson P.. (1998). Isolation of circovirus from lesions of pigs with postweaning multisystemic wasting syndrome. Can. Vet. J. 39, 44–51. PMID: 9442952 PMC1539838

[ref8] Franzo G. Delwart E. Fux R. Hause B. Su S. Zhou J.. (2020b). Genotyping porcine circovirus 3 (PCV-3) nowadays: does it make sense? Viruses 12:265. doi: 10.3390/v12030265 32121102 PMC7150946

[ref9] Franzo G. Ruiz A. Grassi L. Sibila M. Drigo M. Segalés J. (2020a). Lack of porcine circovirus 4 genome detection in pig samples from Italy and Spain. Pathogens 9:6. 10.3390/pathogens9060433 PMC7350368 32486429

[ref10] Hattermann K. Roedner C. Schmitt C. Finsterbusch T. Steinfeldt T. Mankertz A. (2004). Infection studies on human cell lines with porcine circovirus type 1 and porcine circovirus type 2. Xenotransplantation 11, 284–294. doi: 10.1111/j.1399-3089.2004.00134.x, PMID: 15099209

[ref11] Herbst W. Willems H. (2017). Detection of virus particles resembling circovirus and porcine circovirus 2a (PCV2a) sequences in feces of dogs. Res. Vet. Sci. 115, 51–53. doi: 10.1016/j.rvsc.2017.01.014, PMID: 28135670 PMC7111833

[ref12] Hou C. Y. Zhang L. H. Zhang Y. H. Cui J. T. Zhao L. Zheng L. L.. (2022). Phylogenetic analysis of porcine circovirus 4 in Henan Province of China: a retrospective study from 2011 to 2021. Transbound. Emerg. Dis. 69, 1890–1901. doi: 10.1111/tbed.14172, PMID: 34076964

[ref13] Jiang S. Zhou N. Li Y. An J. Chang T. (2019). Detection and sequencing of porcine circovirus 3 in commercially sourced laboratory mice. Vet. Med. Sci. 5, 176–181. doi: 10.1002/vms3.144, PMID: 30779321 PMC6498514

[ref14] Kim D. Y. Kim H. R. Park J. H. Kwon N. Y. Kim J. M. Kim J. K.. (2022). Detection of a novel porcine circovirus 4 in Korean pig herds using a loop-mediated isothermal amplification assay. J. Virol. Methods 299:114350. doi: 10.1016/j.jviromet.2021.114350, PMID: 34748817

[ref15] Kiupel M. Stevenson G. W. Choi J. Latimer K. S. Kanitz C. L. Mittal S. K. (2001). Viral replication and lesions in BALB/c mice experimentally inoculated with porcine circovirus isolated from a pig with postweaning multisystemic wasting disease. Vet. Pathol. 38, 74–82. doi: 10.1354/vp.38-1-74, PMID: 11199167

[ref16] Kiupel M. Stevenson G. Mittal S. Clark E. Haines D. (1998). Circovirus-like viral associated disease in weaned pigs in Indiana. Vet. Pathol. 35, 303–307. doi: 10.1177/030098589803500411, PMID: 9684976

[ref17] Lekcharoensuk P. Morozov I. Paul P. S. Thangthumniyom N. Wajjawalku W. Meng X. J. (2004). Epitope mapping of the major capsid protein of type 2 porcine circovirus (PCV2) by using chimeric PCV1 and PCV2. J. Virol. 78, 8135–8145. doi: 10.1128/JVI.78.15.8135-8145.2004, PMID: 15254185 PMC446101

[ref18] Li L. Kapoor A. Slikas B. Bamidele O. S. Wang C. Shaukat S.. (2010). Multiple diverse circoviruses infect farm animals and are commonly found in human and chimpanzee feces. J. Virol. 84, 1674–1682. doi: 10.1128/JVI.02109-09, PMID: 20007276 PMC2812408

[ref19] Liu Q. Tikoo S. K. Babiuk L. A. (2001). Nuclear localization of the ORF2 protein encoded by porcine circovirus type 2. Virology 285, 91–99. doi: 10.1006/viro.2001.0922, PMID: 11414809

[ref20] Morozov I. Sirinarumitr T. Sorden S. Halbur P. Morgan M. Yoon K.. (1998). Detection of a novel strain of porcine circovirus in pigs with postweaning multisystemic wasting syndrome. J. Clin. Microbiol. 36, 2535–2541. doi: 10.1128/JCM.36.9.2535-2541.1998, PMID: 9705388 PMC105158

[ref21] Mou C. Wang M. Pan S. Chen Z. (2019). Identification of nuclear localization signals in the ORF2 protein of porcine circovirus type 3. Viruses 11:1086. doi: 10.3390/v11121086 31766638 PMC6950156

[ref22] Nayar G. Hamel A. Lin L. (1997). Detection and characterization of porcine circovirus associated with postweaning multisystemic wasting syndrome in pigs. Can. Vet. J. 38, 385–386. PMID: 9187809 PMC1576874

[ref23] Nguyen V. G. Do H. Q. Huynh T. M. Park Y. H. Park B. K. Chung H. C. (2022). Molecular-based detection, genetic characterization and phylogenetic analysis of porcine circovirus 4 from Korean domestic swine farms. Transbound. Emerg. Dis. 69, 538–548. doi: 10.1111/tbed.14017, PMID: 33529468

[ref24] Niu G. Zhang X. Ji W. Chen S. Li X. Yang L.. (2022). Porcine circovirus 4 rescued from an infectious clone is replicable and pathogenic in vivo. Transbound. Emerg. Dis. 69, e1632–e1641. doi: 10.1111/tbed.14498, PMID: 35240007

[ref25] Opriessnig T. Karuppannan A. K. Castro A. Xiao C. T. (2020). Porcine circoviruses: current status, knowledge gaps and challenges. Virus Res. 286:198044. doi: 10.1016/j.virusres.2020.198044, PMID: 32502553

[ref26] Palinski R. Piñeyro P. Shang P. Yuan F. Guo R. Fang Y.. (2017). A novel porcine circovirus distantly related to known circoviruses is associated with porcine dermatitis and nephropathy syndrome and reproductive failure. J. Virol. 91:e01879–16. doi: 10.1128/JVI.01879-16 27795441 PMC5165205

[ref27] Phan T. G. Giannitti F. Rossow S. Marthaler D. Knutson T. P. Li L.. (2016). Detection of a novel circovirus PCV3 in pigs with cardiac and multi-systemic inflammation. Virol. J. 13:184. doi: 10.1186/s12985-016-0642-z 27835942 PMC5105309

[ref28] Shuai J. Wei W. Jiang L. Li X. Chen N. Fang W. (2008). Mapping of the nuclear localization signals in open reading frame 2 protein from porcine circovirus type 1. Acta Biochim. Biophys. Sin. 40, 71–77. doi: 10.1111/j.1745-7270.2008.00377.x, PMID: 18180855

[ref29] Song T. Hao J. Zhang R. Tang M. Li W. Hui W.. (2019b). First detection and phylogenetic analysis of porcine circovirus type 2 in raccoon dogs. BMC Vet. Res. 15:107. doi: 10.1186/s12917-019-1856-2 30961660 PMC6454600

[ref30] Song T. Zhang S. Hao J. Xin S. Hui W. Tang M.. (2019a). First detection and genetic analysis of fox-origin porcine circovirus type 2. Transbound. Emerg. Dis. 66, 1–6. doi: 10.1111/tbed.13004, PMID: 30153367

[ref31] Sun W. Du Q. Han Z. Bi J. Lan T. Wang W.. (2021). Detection and genetic characterization of porcine circovirus 4 (PCV4) in Guangxi, China. Gene 773:145384. doi: 10.1016/j.gene.2020.145384, PMID: 33383119

[ref32] Sun W. Wang W. Xin J. Cao L. Zhuang X. Zhang C.. (2019). An epidemiological investigation of porcine circovirus 3 infection in dogs in the Guangxi Province from 2015 to 2017, China. Virus Res. 270:197663. doi: 10.1016/j.virusres.2019.197663, PMID: 31301332 PMC7114628

[ref33] Tian R. B. Zhao Y. Cui J. T. Zheng H. H. Xu T. Hou C. Y.. (2021). Molecular detection and phylogenetic analysis of porcine circovirus 4 in Henan and Shanxi provinces of China. Transbound. Emerg. Dis. 68, 276–282. doi: 10.1111/tbed.13714, PMID: 32634296

[ref34] Tischer I. Bode L. Apodaca J. Timm H. Peters D. Rasch R.. (1995). Presence of antibodies reacting with porcine circovirus in sera of humans, mice, and cattle. Arch. Virol. 140, 1427–1439. doi: 10.1007/BF01322669, PMID: 7544971

[ref35] Tischer I. Mields W. Wolff D. Vagt M. Griem W. (1986). Studies on epidemiology and pathogenicity of porcine circovirus. Arch. Virol. 91, 271–276. doi: 10.1007/BF01314286 3778212

[ref36] Tischer I. Rasch R. Tochtermann G. (1974). Characterization of papovavirus-and picornavirus-like particles in permanent pig kidney cell lines. Zentralblatt fur Bakteriologie, Parasitenkunde, Infektionskrankheiten und hygiene. Erste Abteilung Originale. Reihe A: Medizinische Mikrobiologie und Parasitologie 226, 153–167. 4151202

[ref37] Turlewicz-Podbielska H. Augustyniak A. Pomorska-Mól M. (2022). Novel porcine circoviruses in view of lessons learned from porcine circovirus type 2-epidemiology and threat to pigs and other species. Viruses 14:2. doi: 10.3390/v14020261 PMC8877176 35215854

[ref38] Vargas-Bermudez D. S. Mogollón J. D. Jaime J. (2022). The prevalence and genetic diversity of PCV3 and PCV2 in Colombia and PCV4 survey during 2015–2016 and 2018–2019. Pathogens 11:6. doi: 10.3390/pathogens11060633 PMC9228467 35745487

[ref39] Wang D. Mai J. Lei B. Zhang Y. Yang Y. Wang N. (2021). Structure, antigenic properties, and highly efficient assembly of PCV4 capsid protein. Front. Vet. Sci. 8:695466. doi: 10.3389/fvets.2021.695466, PMID: 34504886 PMC8421537

[ref40] Wang W. Sun W. Cao L. Zheng M. Zhu Y. Li W.. (2019). An epidemiological investigation of porcine circovirus 3 infection in cattle in Shandong province, China. BMC Vet. Res. 15:60. doi: 10.1186/s12917-019-1793-0 30760271 PMC6375139

[ref41] Wei R. Trus I. Yang B. Huang L. Nauwynck H. (2018). Breed differences in PCV2 uptake and disintegration in porcine monocytes. Viruses 10:10. doi: 10.3390/v10100562 PMC6213064 30326643

[ref42] Wu H. Hou C. Wang Z. Meng P. Chen H. Cao H. (2022). First complete genomic sequence analysis of porcine circovirus type 4 (PCV4) in wild boars. Vet. Microbiol. 273:109547. doi: 10.1016/j.vetmic.2022.109547, PMID: 36037620

[ref43] Xu T. Chen X. M. Fu Y. Ai Y. Wang D. M. Wei Z. Y.. (2022b). Cross-species transmission of an emerging porcine circovirus (PCV4): first molecular detection and retrospective investigation in dairy cows. Vet. Microbiol. 273:109528. doi: 10.1016/j.vetmic.2022.109528, PMID: 35944390

[ref44] Xu T. Hou C. Y. Zhang Y. H. Li H. X. Chen X. M. Pan J. J.. (2022a). Simultaneous detection and genetic characterization of porcine circovirus 2 and 4 in Henan province of China. Gene 808:145991. doi: 10.1016/j.gene.2021.145991, PMID: 34626723

[ref45] Xu T. You D. Wu F. Zhu L. Sun X. G. Lai S. Y.. (2022c). First molecular detection and genetic analysis of porcine circovirus 4 in the southwest of China during 2021-2022. Front. Microbiol. 13:1052533. doi: 10.3389/fmicb.2022.1052533, PMID: 36406418 PMC9668871

[ref46] Zhang D. Bai C. Ge K. Li Y. Gao W. Jiang S.. (2020). Establishment of an SYBR green-based real-time PCR assay for porcine circovirus type 4 detection. J. Virol. Methods 285:113963. doi: 10.1016/j.jviromet.2020.113963, PMID: 32882322

[ref47] Zhang H. H. Hu W. Q. Li J. Y. Liu T. N. Zhou J. Y. Opriessnig T.. (2020). Novel circovirus species identified in farmed pigs designated as porcine circovirus 4, Hunan province, China. Transbound. Emerg. Dis. 67, 1057–1061. doi: 10.1111/tbed.13446, PMID: 31823481

[ref48] Zhang J. Liu Z. Zou Y. Zhang N. Wang D. Tu D.. (2018). First molecular detection of porcine circovirus type 3 in dogs in China. Virus Genes 54, 140–144. doi: 10.1007/s11262-017-1509-0, PMID: 28983774

[ref49] Zhou J. Qiu Y. Zhu N. Zhou L. Dai B. Feng X.. (2021). The nucleolar localization signal of porcine circovirus type 4 capsid protein is essential for interaction with Serine-48 residue of nucleolar phosphoprotein Nucleophosmin-1. Front. Microbiol. 12:751382. doi: 10.3389/fmicb.2021.751382, PMID: 34745055 PMC8566881

