# Supplementary material for: The first dog-origin porcine circovirus type 4 complete genomic sequence have high homology with that of pig-derived strains
Source: Front Microbiol. 2023 Feb 24;14:1121177. doi: 10.3389/fmicb.2023.1121177 (PMC10002969; doi:10.3389/fmicb.2023.1121177)
Supplement: Supplementary file 1 [file Data_Sheet_1.docx]

Table S1 List of primer sequences used in this study

| Primer name | Nucleotide sequence (5’-3’) | Primer locations (bp) | Product size | |
| --- | --- | --- | --- | --- |
| PCV4-1F | GAGGTTCCACCCGTTTAAG | 260-278 | 577 |  |
| PCV4-1R | CCAGTCCTTGATCTGCTTGTTG | 815-836 |  |  |
| PCV4-2F | GCCAAGACAATGTGGATTACC | 792-812 | 690 |  |
| PCV4-2R | AGCCTCCCATTTGCATATTACC | 1460-1481 |  |  |
| PCV4-3F | CCACATAGTCTCCATCCAGTTG | 1361-1382 | 769 |  |
| PCV4-3R | CCCTCCTTTGGAGCAATACTT | 339-359 |  |  |

Table S2 The information of all strains for sequence alignment and phylogenetic analysis

| Strain | Organism | Size | Collection date | Country | Accession number | Strain | Organism | Size | Collection date | Country | Accession number |
| --- | --- | --- | --- | --- | --- | --- | --- | --- | --- | --- | --- |
| HNU-AHG1-2019 | Porcine circovirus 4 | 1770 | Feb-2019 | China | MK986820.1 | HN-ZK-201707 | Porcine circovirus 4 | 1770 | Jul-2017 | China | MW600960.1 |
| Henan-LY1-2019 | Porcine circovirus 4 | 1770 | Feb-2019 | China | MT015686.1 | HN-LY-202005 | Porcine circovirus 4 | 1770 | May-2020 | China | MW538943.1 |
| KF-02-2019 | Porcine circovirus 4 | 1770 | Oct-2019 | China | MT193105.1 | HN-LY-202006 | Porcine circovirus 4 | 1770 | Jun-2020 | China | MW600947.1 |
| KF-01-2019 | Porcine circovirus 4 | 1770 | Oct-2019 | China | MT193106.1 | HN-LY-202007 | Porcine circovirus 4 | 1770 | Jul-2020 | China | MW600948.1 |
| PCV4/GX2020/NN88 | Porcine circovirus 4 | 1770 | 2018 | China | MT311852.1 | HN-SMX-202011 | Porcine circovirus 4 | 1770 | Nov-2020 | China | MW600949.1 |
| PCV4/GX2020/GL69 | Porcine circovirus 4 | 1770 | 2018 | China | MT311853.1 | HN-XX-201811 | Porcine circovirus 4 | 1770 | Nov-2018 | China | MW600950.1 |
| PCV4/GX2020/FCG49 | Porcine circovirus 4 | 1770 | 2018 | China | MT311854.1 | HN-KF-201812 | Porcine circovirus 4 | 1770 | Dec-2018 | China | MW600951.1 |
| FJ-PCV4 | Porcine circovirus 4 | 1770 | 2019 | China | MT721742.1 | HN-HB-201704 | Porcine circovirus 4 | 1770 | Apr-2017 | China | MW600952.1 |
| JSYZ1901-2 | Porcine circovirus 4 | 1770 | 02-Jan-2019 | China | MT769268.1 | HN-XX-201212 | Porcine circovirus 4 | 1770 | Dec-2012 | China | MW600953.1 |
| E115 | Porcine circovirus 4 | 1770 | 23-Apr-2020 | South Korea | MT882344.1 | HN-LY-201702 | Porcine circovirus 4 | 1770 | Feb-2017 | China | MW600954.1 |
| PCV4/CN/NM1/2017 | Porcine circovirus 4 | 1770 | 2017 | China | MT882410.1 | HN-ZZ-201603 | Porcine circovirus 4 | 1770 | Mar-2016 | China | MW600955.1 |
| PCV4/CN/NM2/2017 | Porcine circovirus 4 | 1770 | 2017 | China | MT882411.1 | HN-ZK-201512 | Porcine circovirus 4 | 1770 | Dec-2015 | China | MW600956.1 |
| PCV4/CN/NM3/2017 | Porcine circovirus 4 | 1770 | 2017 | China | MT882412.1 | HN-ZK-201601 | Porcine circovirus 4 | 1770 | Jan-2016 | China | MW600957.1 |
| Hebei-AP1-2019 | Porcine circovirus 4 | 1770 | 2019 | China | MW084633.1 | HN-ZMD-201212 | Porcine circovirus 4 | 1770 | Dec-2012 | China | MW600958.1 |
| Hebei1 | Porcine circovirus 4 | 1770 | 10-Sep-2020 | China | MW262973.1 | HN-XX-201601 | Porcine circovirus 4 | 1770 | Jan-2016 | China | MW600959.1 |
| Hebei2 | Porcine circovirus 4 | 1770 | 15-Sep-2020 | China | MW262974.1 | KU-02011 | Porcine circovirus 4 | 1770 | Nov-2020 | South Korea | MW712667.1 |
| Hebei3 | Porcine circovirus 4 | 1770 | 15-Sep-2020 | China | MW262975.1 | KU-02010 | Porcine circovirus 4 | 1770 | Oct-2020 | South Korea | MW712668.1 |
| Hebei4 | Porcine circovirus 4 | 1770 | 20-Sep-2020 | China | MW262976.1 | PCV4-YY2019 | Porcine circovirus 4 | 1770 | 2019 | China | MW759027.1 |
| Hebei5 | Porcine circovirus 4 | 1770 | 20-Sep-2020 | China | MW262977.1 | PCV4-HB2017 | Porcine circovirus 4 | 1770 | 2017 | China | MW759028.1 |
| Hebei6 | Porcine circovirus 4 | 1770 | 20-Sep-2020 | China | MW262978.1 | PCV4-LY2017 | Porcine circovirus 4 | 1770 | 2017 | China | MW759029.1 |
| Hebei-Rac1 | Porcine circovirus 4 | 1770 | 01-Oct-2015 | China | MW262979.1 | JXWY-2021 | Porcine circovirus 4 | 1770 | 2020 | China | MW988108.1 |
| Hebei-Rac2 | Porcine circovirus 4 | 1770 | 07-Nov-2017 | China | MW262980.1 | JXSC-2021 | Porcine circovirus 4 | 1770 | 2020 | China | MW988109.1 |
| Hebei-Rac3 | Porcine circovirus 4 | 1770 | 16-Jun-2019 | China | MW262981.1 | SC-GA2022ABTC | Porcine circovirus 4 | 1770 | 2022 | China | OP497960.1 |
| Hebei-Rac4 | Porcine circovirus 4 | 1770 | 13-Jun-2018 | China | MW262982.1 | PCV4-LY2020 | Porcine circovirus 4 | 1770 | 2020 | China | MW759026.1 |
| Hebei-Rac5 | Porcine circovirus 4 | 1770 | 02-Jun-2018 | China | MW262983.1 | FJ2020001 | Porcine circovirus 4 | 1770 | Jan-2020 | China | MW238796.1 |
| Hebei-Fox1 | Porcine circovirus 4 | 1770 | 25-Jun-2018 | China | MW262984.1 | SCABTC-Dog2022 | Porcine circovirus 4 | 1770 | 2022 | China | OP948894 |

Note: UN, Unknow.

Note: three accession numbers (APG55803, ASH99030 and AXK90322) labeled with※ were the amino acid sequences of Rep, and their “size” represents the length of the amino acid. Other accession numbers represent nucleotide sequences.
